# Evaluation of Influence of Single Nucleotide Polymorphisms in Cytochrome P450 2B6 on Substrate Recognition Using Computational Docking and Molecular Dynamics Simulation

**DOI:** 10.1371/journal.pone.0096789

**Published:** 2014-05-05

**Authors:** Kana Kobayashi, Ohgi Takahashi, Masahiro Hiratsuka, Noriyuki Yamaotsu, Shuichi Hirono, Yurie Watanabe, Akifumi Oda

**Affiliations:** 1 Faculty of Pharmaceutical Sciences, Tohoku Pharmaceutical University, Aoba-ku, Sendai, Miyagi, Japan; 2 Graduate School of Pharmaceutical Sciences, Tohoku University, Aoba-ku, Sendai, Miyagi, Japan; 3 School of Pharmacy, Kitasato University, Minato-ku, Tokyo, Japan; 4 Faculty of Pharmacy, Institute of Medical, Pharmaceutical and Health Sciences, Kanazawa University, Kanazawa, Ishikawa, Japan; 5 Institute for Protein Research, Osaka University, Suita, Osaka, Japan; German Research School for Simulation Science, Germany

## Abstract

In this study, we investigated the influence of single nucleotide polymorphisms on the conformation of mutated cytochrome P450 (CYP) 2B6 proteins using molecular dynamics (MD) simulation. Some of these mutations influence drug metabolism activities, leading to individual variations in drug efficacy and pharmacokinetics. Using computational docking, we predicted the structure of the complex between the antimalarial agent artemether and CYP2B6 whose conformations were obtained by MD simulation. The simulation demonstrated that the entire structure of the protein changes even when a single residue is mutated. Moreover, the structural flexibility is affected by the mutations and it may influence the enzyme activity. The results suggest that some of the inactive mutants cannot recognize artemether due to structural changes caused by the mutation.

## Introduction

Cytochrome P450 (CYP) belongs to the superfamily of heme proteins [1]–[5]. The active site with the prosthetic iron group is located in the central portion of the heme protein, whose molecular weight is approximately 50,000 [1]. Within the cell, CYP has mainly been found in the microsome. CYPs are widely distributed from bacteria to plants and animals. Although CYP was first discovered in the animal liver, many studies have since revealed that various CYPs are found in almost every animal tissue.

CYP catalyzes monooxygenase reactions of endogenous and exogenous compounds. For example, CYP synthesizes and metabolizes physiological active substances such as steroid hormones and fat soluble vitamins and metabolizes medicine and environmental pollutants. Drug metabolic reactions are classified into two groups: phase I includes oxidation, reduction, and hydrolysis reactions and phase II includes conjugation reactions. CYP is mostly involved in oxidation reactions of phase I metabolism [2]. The mechanism through which CYP participates in oxidation reactions has been investigated not only by experiments, but also by computational methods [3], [5].

Many CYPs are major enzymes involved in drug metabolism, i.e., the chemical modification or degradation of drugs. In humans, drug-metabolizing CYPs include CYP1A2, CYP2B6, CYP2C9, CYP2C19, CYP2D6, and CYP3A4 among others. Most CYPs display low substrate specificity: one CYP frequently metabolizes several substrates and one substrate can be metabolized by several CYPs. In contrast, some drugs are metabolized by a single CYP. In the latter case, the absence of the CYP species causes side effects because the drug cannot be metabolized and remains in the body. Molecular species of human drug-metabolizing CYP have many genetic variants. This genetic polymorphism influences drug metabolism and causes individual variation of drug efficacies and side effects, increasing the importance of tailor-made medicines [6].

**Figure 1:**
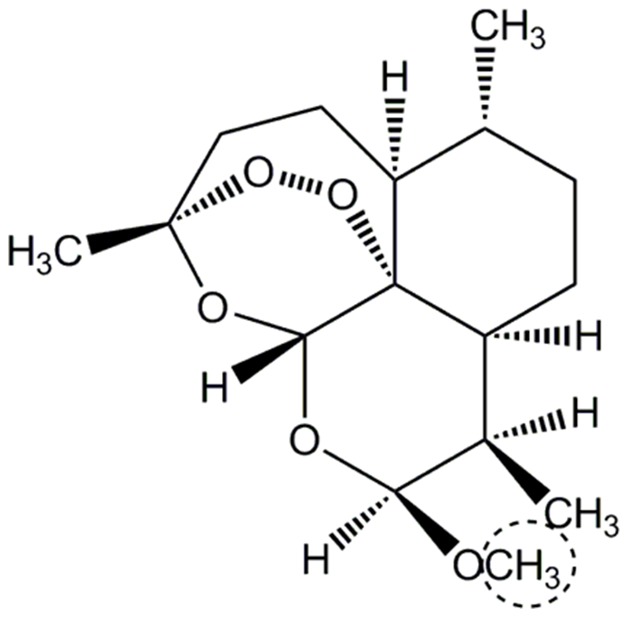
Structure of AM. The position demethylated by CYP is indicated by a dashed line.

Artemether (AM) (Fig. 1) is recommended as first-line treatment for malaria because it is a rapid acting drug and no serious side effect has been reported to date. AM is an artemisinin derivative containing a sesquiterpene lactone moiety [7]. AM is considered to be mainly metabolized by CYP2B6 and CYP3A4. A study using human liver microsomal fractions [8] showed that CYP2B6 has a more important role in the demethylation of AM than CYP3A4. By the elimination of the methyl group, AM is metabolized into dihydroartemisinin (DHA), which has a higher antimalarial activity than AM. Both AM and DHA have a very short and variable half-life (1 hour for AM; 45 minutes for DHA), and their blood concentration varies depending on individual and racial differences [9].

The CYP2B6 protein (491 amino acids) belongs to the CYP2B subfamily and is found in several organs, including the liver, lung, and gastrointestinal tract. Although the CYP2 family includes the largest number of molecular species in CYP, their amino acid sequences in the substrate recognition site are poorly conserved [10].

The expression ratio of CYP2B6 compared with CYP is 1%–6%, as detected by antibody-based expression analysis [11], [12]. CYP2B6 contributed to 7.6% of drug metabolism in a study that compared more than 1300 CYP-related substances including drugs and other compounds [13]. Although the contribution of CYP2B6 is 7.6%, more than 70 substrates are metabolized by CYP2B6, ranging from the antimalarial drug AM to the anticancer drug cyclophosphamide, the anti-HIV drug efavirenz, and the antidepressant and smoking-cessation drug bupropion. Thus, CYP2B6 is an important enzyme in drug metabolism. More than 30 variants have been reported for CYP2B6, and this genetic polymorphism is regarded as important [14].

We previously measured *in vivo* activities of the wild-type and mutants of CYP2B6 toward AM demethylation [8]. Our study indicates that CYP2B6.2, CYP2B6.4, CYP2B6.6, and CYP2B6.19 have a higher activity than the wild-type, whereas CYP2B6.10, CYP2B6.11, CYP2B6.14, CYP2B6.15, CYP2B6.16, CYP2B6.20, and CYP2B6.27 have a lower activity than the wild-type. Moreover, no activity was detectable for CYP2B6.8, CYP2B6.12, CYP2B6.18, CYP2B6.21, or CYP2B6.24; these five mutants are single nucleotide polymorphisms (SNPs).

In general, the three-dimensional (3D) structures of enzymes and enzyme–substrate complexes are necessary to understand the mechanisms of drug metabolism from the structural biology point of view. Five structures of the CYP2B6–ligand complex are available in the Protein Data Bank (PDB). The ligands in these structures are either rigid, small compounds (PDB ID: 3IBD, 3QOA, 3QU8, and 4I91) or a calcium channel blocker with a flexible side chain (3UA5). All five PDB entries used the same CYP2B6 artificial mutant (Y226H/K262R). Therefore, no structure of native CYP2B6 bound to AM, or a similar large compound is available. Computational studies have investigated the substrate recognition of CYP2B6 at the atomic level. Niu et al. performed a 300-ps molecular dynamics (MD) simulation of CYP2B6 and computational docking between CYP2B6 and ligands such as efavirenz and bupropion [15]. The study indicates that mainly Phe297, Glu301, Thr302, and Val367 have important roles in drug recognition by CYP2B6. Bathelt et al. conducted docking studies between CYP2B6 and cyclophosphamide to investigate the structural features of the substrate-binding site of CYP2B6 [16]. These studies have provided an insight into substrate recognition by CYP2B6. However, Niu et al. constructed the CYP2B6 structure by removing an inhibitor 4-(4-chlorophenyl) imidazole from 3IBD, and Bathelt et al. generated the CYP2B6 structure by homology modeling from CYP2B5 using the Swiss-Model. Thus, there are no studies dealing explicitly with polymorphism. On the other hand, MD simulations were performed to evaluate the effects of genetic polymorphism in other CYP2 molecular species. Oda et al. previously predicted the 3D structures of the wild-type and mutants of CYP2C19, and investigated their recognition mechanism [17]. The results suggest that the hydrogen bond between the substrate (S)-mephenytoin and CYP2C19 detected in the wild-type cannot form in mutants, which were known to be inactive. To investigate the difference of substrate specificity between the wild-type and mutants of CYP1A2, MD simulation of the complex structure between CYP1A2 and the substrate was performed [18]. Because each molecular species has generally several variants, it is extremely difficult to obtain 3D structures of all variants experimentally. Moreover, substrate recognition mechanisms of all variants cannot be experimentally elucidated at the atomic level. On the other hand, computational methods can reveal the structural features of several CYPs as mentioned above [17], [18]. Although the simulation of the natural mutant of CYP2B6 has not been performed yet, structural bioinformatics may be useful for investigating the influence of polymorphism on 3D structures for CYP2B6 similar to other CYPs.

In this study, we performed MD simulations to elucidate the effect of SNPs on the 3D structure of CYP2B6. A total of eight mutants were used in this study: five SNPs were inactive, one was an enhanced activity mutant, and two were lower activity mutants. We attempted to perform longer MD simulations than those performed in previous studies [15], [16] to discuss the change in 3D structures caused by mutations. Finally, we evaluated the effects of polymorphism on ligand recognition using docking studies of AM with the wild-type and mutants of CYP2B6 whose 3D structures were obtained from MD simulation.

## Methods

First, we investigated the effects of amino acid mutations on the 3D structures using minimization and MD simulation of the wild-type and mutants of CYP2B6. 3D structures of CYP2B6 were constructed from experimentally determined structure retrieved from the PDB (PDB ID: 3IBD [19]). The inhibitor 4-(4-chlorophenyl) imidazole was eliminated from 3IBD. Then, because 3IBD includes two mutations, Y226H and K262R, these mutants were replaced by wild-type residues to construct the CYP2B6.1 model structure. The structural refinement of CYP2B6.1 was performed by MD simulation. From the refined structure of CYP2B6, 3D structures of eight mutants were generated, and the structural refinement of each mutant was performed by MD simulation. The wild-type and mutants used in this study are presented in Table 1. The computational results described below are also shown in Table 1. The initial 3D structures of CYP2B6.1, CYP2B6.12, CYP2B6.15 and CYP2B6.21 were evaluated with Verify3D [20]. To investigate the structural features of the wild-type and some mutants, ligand-binding pockets of proteins were detected with HBOP and HBSITE [21], [22].

**Table 1:** Mutation and activity of the wild-type and mutants of CYP2B6 used in this study.

	mutation	activity[8]	MD not converged	RMSF different from wild-type	appropriate pose not obtained
CYP2B6.1	wild-type	wild-type		–	
CYP2B6.4	Lys262Arg	increase			
CYP2B6.8	Lys139Glu	N.D.		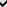 ^a,b)^	
CYP2B6.11	Met46Val	decrease		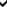 ^b)^	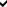
CYP2B6.12	Gly99Glu	N.D.	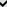	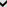 ^b,c)^	
CYP2B6.15	Ile391Asn	decrease	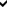	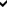 ^b)^	
CYP2B6.18	Ile328Thr	N.D.		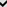 ^b)^	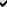
CYP2B6.21	Pro428Thr	N.D.	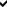	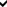 ^c,d)^	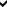
CYP2B6.24	Gly476Asp	N.D.		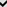 ^c)^	

N.D.: not detectable.

a) RMSFs around C helices are large.

b) RMSFs around G helices are small.

c) RMSFs around I helices are large.

d) RMSFs around L helices are large.

MD simulations were performed under constant pressure, and a temperature-increase MD was performed before equilibrating MD. In temperature-increase MD, the simulation temperature was raised from 0 K to 300 K. Subsequent equilibrating MD was performed at 300 K. TIP3P explicit water solvents were used and calculated under cyclic-boundary conditions. The cutoff distance for van der Waals interactions was 10 Å, and the MD simulation was performed using the particle-mesh Ewald method for calculating electrostatic interactions. The size of cyclic-boundary boxes was determined by the protein size with at least an 8-Å margin, and TIP3P water molecules filled the box. The chlorine ions were used as counter-ions for neutralizing the systems. The force field parameters around the heme iron determined by us [23] were used, and parameters determined by Giammona were used for the rest of the heme moiety. Our parameters were for sextet state with five-coordinate iron (III), and the spin and coordination states of CYP2B6 were the same as those of the ref. [23] in this study. In the catalytic cycle of CYP, substrates are recognized by this state of CYP. We used the AMBER ff99SB force field for amino acids [24], [25]. 20 ps (20,000 steps) calculations were carried out for temperature-increase MD and 20 ns (20,000,000 steps) calculations for equilibrating MD. In actual drug design trials, around 10 ns or tens of nanoseconds MD simulations are generally carried out [26], [27], therefore, we performed 20 ns MD simulations for the drug-related protein CYP2B6 to imitate the actual drug design. Amber10 was used for minimizations and MD simulations [28]. The tleap module of AmberTools was used for building the mutants.

After the 20-ns MD simulations, docking studies between CYP2B6 (wild-type or mutants) and the antimalarial drug AM were performed. The 3D structure of AM was optimized by the hybrid density functional theory (DFT) B3LYP/6-31+G (df,p) using Spartan'08 [29]. The optimized structure of AM was docked to wild-type and eight mutants. The docking studies were performed by the LibDock program [30]. For the docking studies, ligand binding sites were explored, and the nearest site to the heme was used because heme is the active center of CYP. The radius of the site was set to 15 Å. The “Conformation method” was changed from FAST to BEST and the number of hotspots was set to 1,000 for the elaborate calculations. If the 10-methoxy group of AM was directed at the heme iron in the predicted complex structure, the ligand pose was defined as a “reasonable pose” because of the catalytic mechanism of AM by CYP2B6. For the 3D structures of complexes obtained by docking, minimizations were performed using the same settings described above.

## Results and Discussion

**Figure 2:**
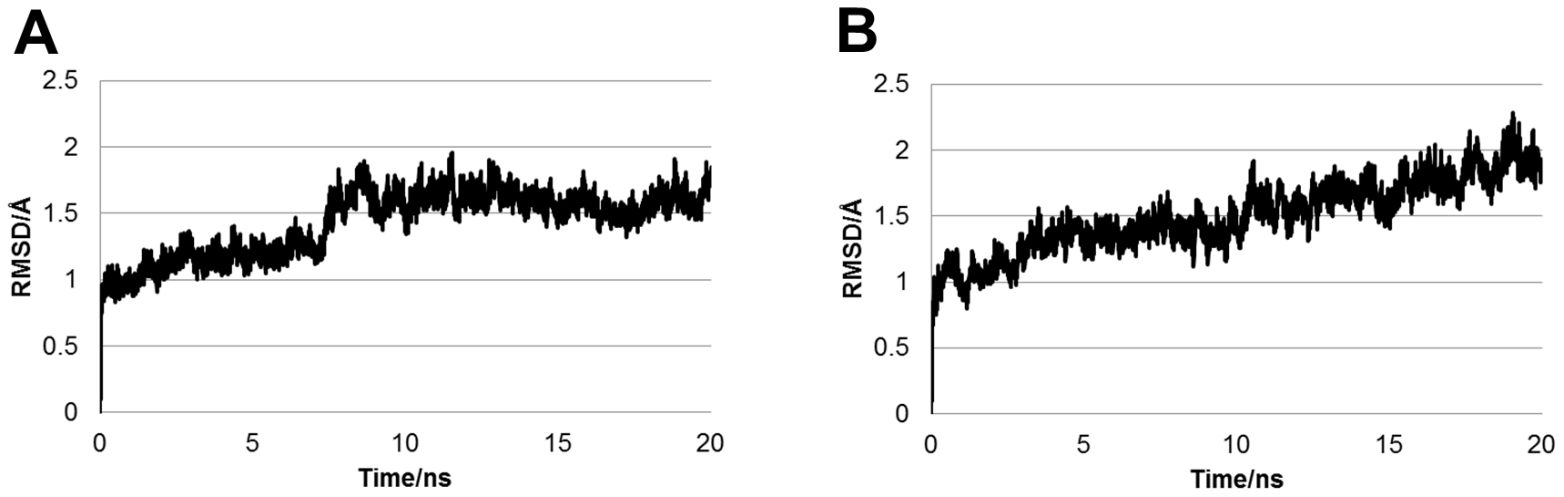
RMSDs for two mutants of CYP2B6. RMSD of CYP2B6.4 (A) converged, whereas that of CYP2B6.12 (B) did not converge at 20 ns.

In this study, a wild-type structure was constructed from the PDB entry (3IBD). Therefore, the constructed 3D structure of wild-type was validated with Verify3D. For comparison, the crystal structure 3IBD was also validated. The score calculated with Verify3D were comparable between the constructed wild-type structure and crystal structure, and the average scores for the wild-type and crystal structure residues were 0.463 and 0.449, respectively. This result indicates that the constructed 3D structure of wild-type is equally reliable as the original crystal structure. Subsequently, to validate the MD simulations, the root mean square deviations (RMSDs) for main chain atoms of the wild-type and mutants along the MD trajectory were calculated. For the RMSD calculations of the wild-type, the initial structure before minimization was used as the reference structure. For the RMSD calculations of the mutants, the structure of the wild-type after 5-ns MD simulation was used as reference. Because the RMSD of CYP2B6.1 converged at approximately 1 ns, the result indicates that the equilibrium structure of CYP2B6.1 was obtained by the MD simulation. The MD simulations of CYP2B6.4, CYP2B6.8, CYP2B6.11, CYP2B6.18, and CYP2B6.24 converged at 20 ns; thus, equilibrium structures of these mutants can also be obtained. On the other hand, the RMSDs of other mutants increased continuously by 20-ns MD simulation, suggesting that the mutations cause a conformational change in CYP2B6 not only locally but also globally, despite a single-residue mutation. RMSDs for the two mutants CYP2B6.4 and CYP2B6.12 are shown in Fig. 2. RMSD of CYP2B6.4 converged at approximately 10 ns, and the equilibrium 3D structure was obtained (Fig. 2(a)); however, RMSD of CYP2B6.12 had not converged even at 20 ns (Fig. 2(b)). The initial structures of CYP2B6.12, CYP2B6.15, and CYP2B6.21, which did not converge at 20 ns, were evaluated with Verify3D. Their calculated scores were comparable to those of the crystal and wild-type structures, and the averages of the scores for CYP2B6.12, CYP2B6.15, and CYP2B6.21 residues were 0.435, 0.434, and 0.435, respectively. In addition, the scores for each CYP2B6.12, CYP2B6.15, and CYP2B6.21 residues were similar to those of the crystal and wild-type structures. To investigate whether the MD results depended on the initial structures, MD simulations were performed for CYP2B6.12, CYP2B6.15, and CYP2B6.21 using other initial structures that were constructed from crystal structures other than 3IBD, whose PDB ID was 4I91. After the 3D structures of CYP2B6.12, CYP2B6.15, and CYP2B6.21 were constructed from 4I91, the 20-ns MD simulations were performed. The MD simulation for CYP2B6.12 converged at 20 ns, suggesting that the MD simulation was influenced by the initial structure. In contrast, MD simulations for CYP2B6.15 and CYP2B6.21 did not converge at 20 ns, regardless of whether 4I91 was used to construct the initial structures. In the mutants whose MD simulations did not converge, the global conformational changes may interfere with substrate recognition by CYP2B6 and reduce the metabolism activity. CYP2B6.21 is the only mutant in which the mutation site is located near the heme, and the distance between the mutated amino acid Thr428 and the heme iron is approximately 7 Å. For the other mutants, the mutated amino acids were away from the heme. The experimental observations that the activity of these mutants decreases or becomes null support the computational result that the single residue mutation described above (in addition, away from the active site) can influence the conformation of the whole protein.

**Figure 3:**
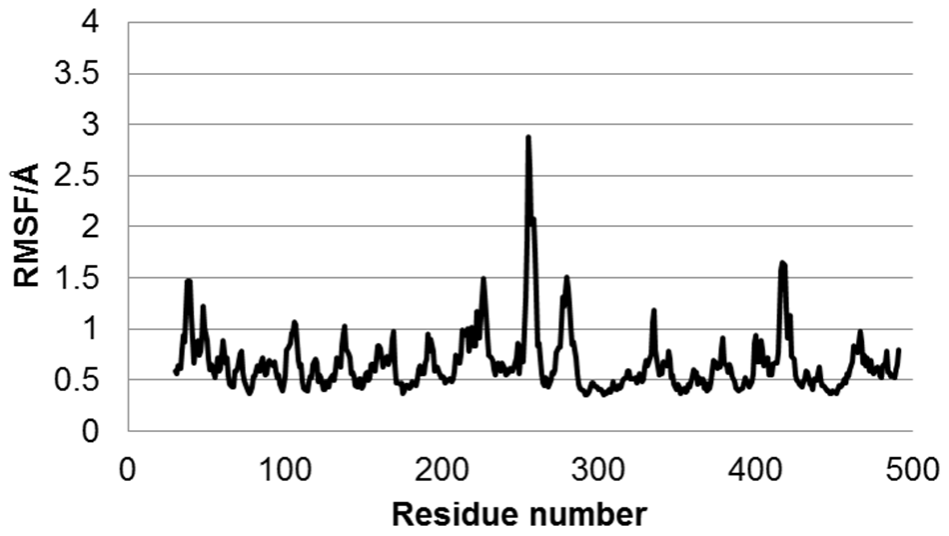
RMSF of the Cα atoms in the wild-type. The highest peak was Thr255. Around the highest peak, there are RMSF peaks at those for Tyr226 and Ala279. An additional peak is observed at the peak for Asn417.

**Figure 4:**
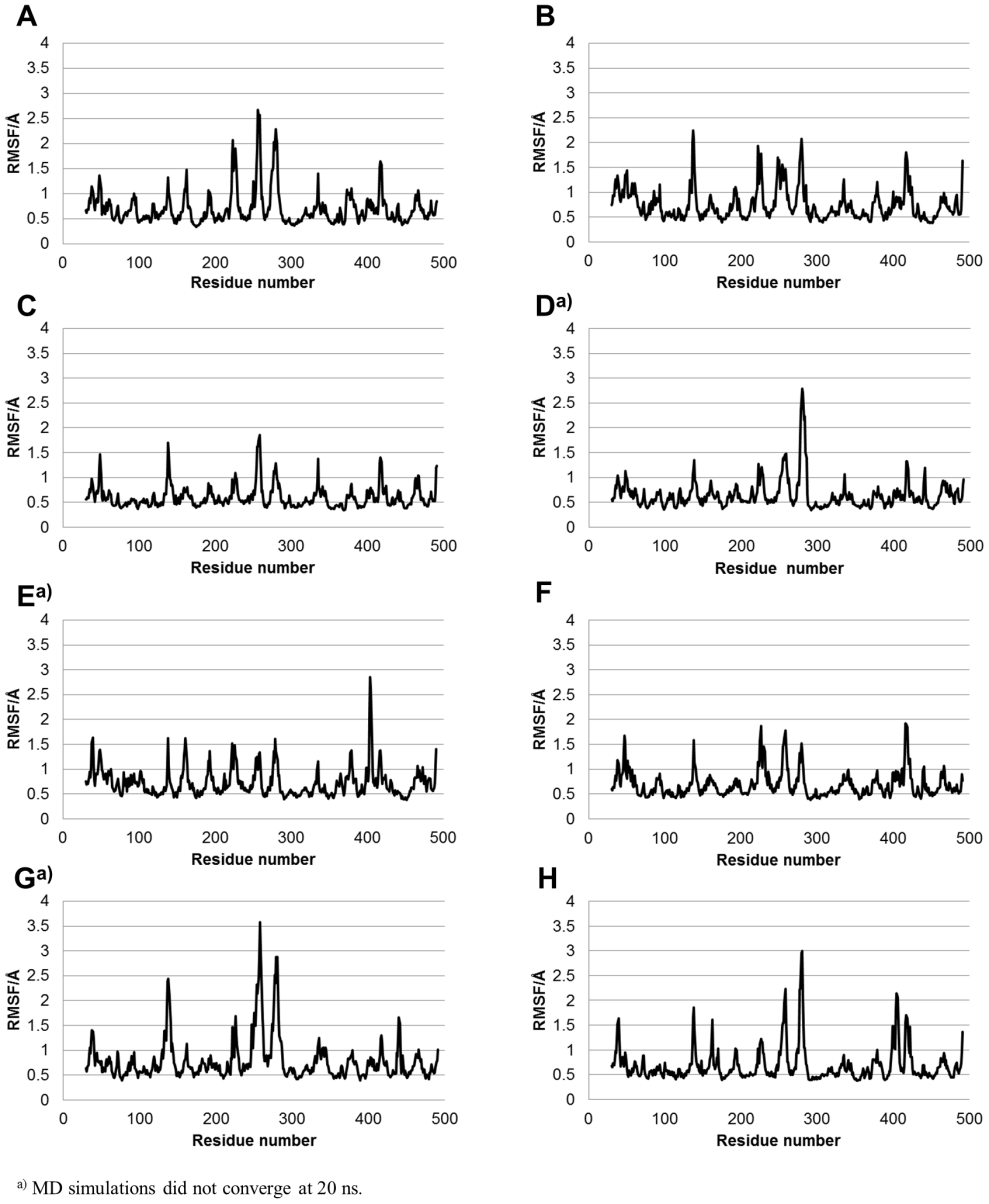
RMSFs of the Cα atoms in mutants. (A) CYP2B6.4, (B) CYP2B6.8, (C) CYP2B6.11, (D) CYP2B6.12, (E) CYP2B6. 15, (F) CYP2B6. 18, (G) CYP2B6.21, and (H) CYP2B6.24. In some mutants, the locations and heights of the peaks are different from those in the wild-type. A prominent peak is observed at 100–200th residues in some mutants but not in the wild-type.

To clarify the residues that are flexible, root mean square fluctuations (RMSF) were calculated using MD trajectories. The average structures during the last 5 ns of the 20-ns MD simulations were used as the reference structure to calculate the RMSFs, and the fluctuations in the last 5 ns were calculated. The effects of translation and rotation were eliminated for the RMSF calculations. The RMSF of the wild-type are shown in Fig. 3. The residue number is shown in the abscissa axis, and the RMSF for the Cα atom of each residue is in the ordinate axis. Moreover, the RMSFs of mutants are shown in Fig. 4. The highest peaks of RMSF for CYP2B6.4, CYP2B6.11, and CYP2B6.21 were located near that of RMSF for CYP2B6.1; however, for the other mutants, the highest peaks were different from that for CYP2B6.1. This suggests that the flexibilities of some mutant proteins are dominantly different from the wild-type despite their structural similarity to the wild-type (RMSDs of every mutant was approximately 2.0 Å). Although the location of the highest peak for CYP2B6.11 and CYP2B6.21 were similar, the overall RMSF of CYP2B6.11 was lower and that of CYP2B6.21 was higher than that of the wild-type. The differences in protein fluctuation may influence the flexibility for substrate recognition and the interaction with the ligand, and further influence the enzyme activities of CYP2B6 mutants. As shown in Table 1 and Figs. 3 and 4, the sites in which RMSFs were largely different between the wild-type and mutants were not always located near the mutated amino acid. For example, RMSFs of the 250–300th residues of CYP2B6.12 were largely different from those of the wild-type, although the mutation of CYP2B6.12 is Gly99Glu. Therefore, the mutation influences not only the structures of adjacent residues but also those of distant residues.

CYP consists of α-helix-rich and β-sheat-rich regions; CYP2B6 has 21 α-helices. The heme in the active site is surrounded by the C, I, and L helices. In addition, the G helix is located near the highest peak (Thr255) of the RMSF in the wild-type. Therefore, we focused on the C, I, L, and G helices.

**Figure 5:**
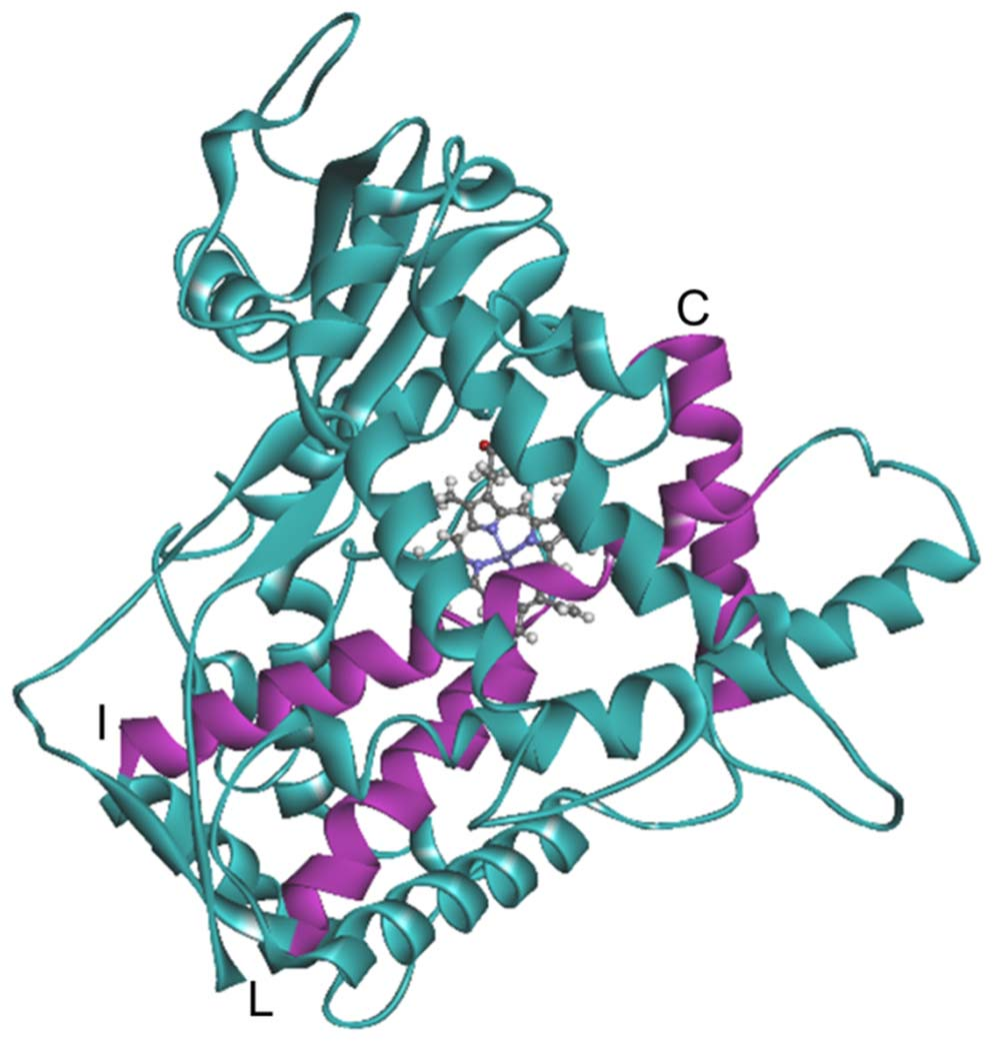
The structure of CYP2B6 shown by a ribbon diagram. The helices near the substrate recognition site, C, I, and L helices, are shown in purple.

**Figure 6:**
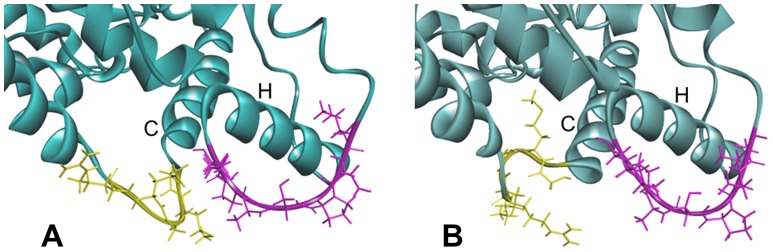
Ribbon-and-stick diagram showing the C/D loop (yellow) and the G/H loop (purple). (A) CYP2B6.8. (B) CYP2B6.11. The relative position of the C/D and the G/H loop differs dramatically among mutants.

The C helix consists of the amino acids from Asn117 to Phe135 and is located close to the heme plane as shown in Fig. 5. RMSFs of the residues near the C helix in the mutants, except CYP2B6.4 and CYP2B6.12, increased in comparison with those in the wild-type. In CYP2B6.8 and CYP26.15, the distance between the C/D loop and the G/H loop was less than 3 Å, which is closer than the distance of the wild-type (about 5 Å). The conformational change was particularly remarkable in CYP2B6.8 because the mutation site is located on the C/D loop region and the mutation Lys139Glu substitutes a basic amino acid for an acidic one. On the contrary, the 3D structure of the C/D loop in CYP2B6.11 was different from the wild-type and the distance between the C/D loop and the G/H loop increased (Fig. 6). As discussed later, the G/H loop is considered a key portion for the CYP enzyme activity. Thus, the structural flexibility of the C/D loop may affect the structure of the G/H loop, and the structural change of the G/H loop may lead to changes in the enzyme activity of mutants.

The highest peak of RMSF for the wild-type Thr255 is located in the C terminal of the G helix. This helix is exposed on the protein surface, and Thr255 is at the boundary region of the helix and the flexible loop structure. The G helix and the subsequent H helix are structurally flexible and the movement of these portions has important roles in the recognition of substrates and the redox partner [19]. We found that this peak was smaller in some mutants than in the wild-type. This was particularly the case for CYP2B6.12 and CYP2B.15, where the lower RMSF peak of this position implies that this portion is rigid in these mutants. The lower flexibility of the G/H loop, considered as the important region for the ligand recognition, may prevent AM from binding and explain the reduced activity of these mutants.

**Figure 7:**
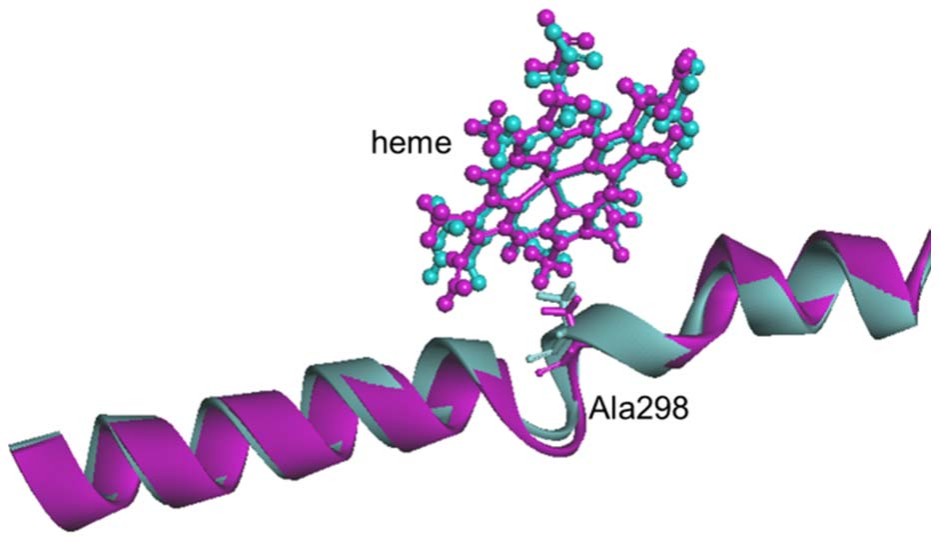
Ribbon-and-stick diagram showing the I helix and the heme of the CYP2B6 wild-type (blue) and CYP2B6.12 variant (purple).

I helix, the longest α-helix in CYP, consists of amino acids from Asn287 to Tyr317 and is part of the substrate binding pocket of CYP. This helix is considered as a key portion for CYP enzyme activity similar to the G/H loop [31]. A highly conserved threonine residue, located in the middle of the I helix, induces a bending, extending slightly the space between the I helix and the heme. This threonine is involved in the binding of oxygen to iron. All mutants except CYP2B6.11 had a peak of RMSF around the I helix, with higher peaks observed for CYP2B6.12, CYP2B6.21, and CYP2B6.24. In CYP2B6.12, the I helix was the most distorted compared with the wild-type, and the distance is increased between the heme and Ala298, the closest amino acid to the heme in the I helix (Fig. 7).

In the L helix, consisting of amino acids from Gly438 to Asn456, only CYP2B6.21 showed a significant peak of RMSF at approximately 1.7 Å. Cys436, which is the vertical ligand of the heme iron, is located at the N-terminal side of this rigid helix. The mutation site of CYP2B6.21, Pro428Thr, which is eight amino acids away, affects the location of Cys436 between the K''' and L helices. Because the heme iron forms the coordinate bond with the sulfur of Cys436, the flexibility of the heme may be influenced by the Pro428Thr mutation.

**Figure 8:**
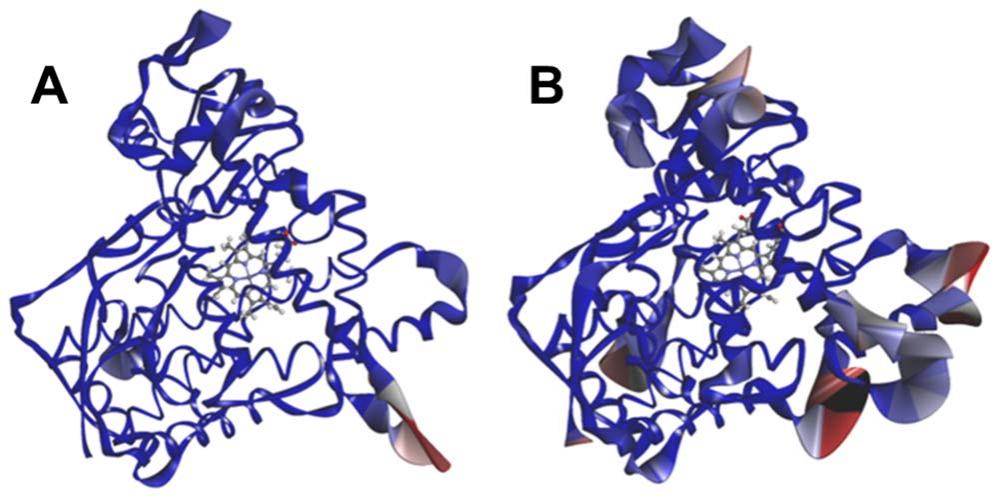
CYP2B6.1 and CYP2B6.8 colored for isotropic displacement. The larger value of the isotropic displacement is indicated by red.

In CYP2B6.8, the flexibility of the whole structure is increased as several peaks of approximately 2 Å are present. However, the highest peak in the wild-type around G helix was low in CYP2B6.8, indicating that this part of the structure is rigid in the mutant (Fig. 8). As indicated above, in CYP2B6.21, the peaks of RMSF were higher than those of the wild-type not only for the C helix region but also for the C terminal part of the G helix, around the I helix and an additional peak around the L helix. These data suggest that CYP2B6.21 is more flexible than CYP2B6.1. Furthermore, many mutants and the wild-type had peaks from the 400th to the 430th amino acids; flexibility in this part is expected because it is mainly composed of structures exposed at the surface of the protein. In particular, the highest peak of RMSF was observed for CYP2B6.15, possibly due to the mutation of Ile391 located in the region adjacent to the loops.

**Figure 9:**
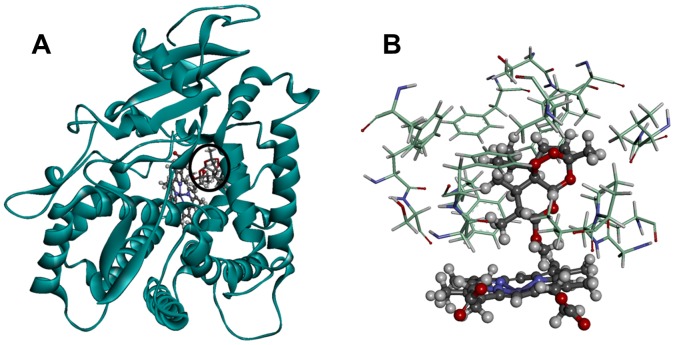
Structure of the complex between the wild-type and AM after docking simulation. (A) The whole complex structure. CYP2B6.1 is shown by ribbon, heme and AM are shown by ball-and-stick representation. AM is circled. (B) Structure around the active site. The residues of CYP2B6.1 are shown by stick, heme and AM are shown by ball-and-stick representation.

The complex structures of CYP2B6 with AM were predicted by computational docking for the wild-type and mutants. The docking poses were evaluated with reference to the metabolism of AM by CYP2B6. Because the demethylation of AM is catalyzed by CYP2B6, the docking poses for which the demethylation position shown in Fig. 1 is close to the heme iron is defined as an “appropriate pose.” The predicted structure of the complex between the wild-type CYP2B6 and AM in which the distance between the demethylation site of AM and the heme iron was the shortest is shown in Fig. 9. Because 10-methoxy group of AM is close to the heme iron in the predicted structure of the complex shown in Fig. 9, the catalytic reaction is expected to occur in the 10-position of AM. The expected reaction product from the *O*-alkyl group closest to the heme iron of CYP is the *O*-dealkylation compound, dihydroartemisinin. Therefore, the docking results were consistent with experimental data.

**Figure 10:**
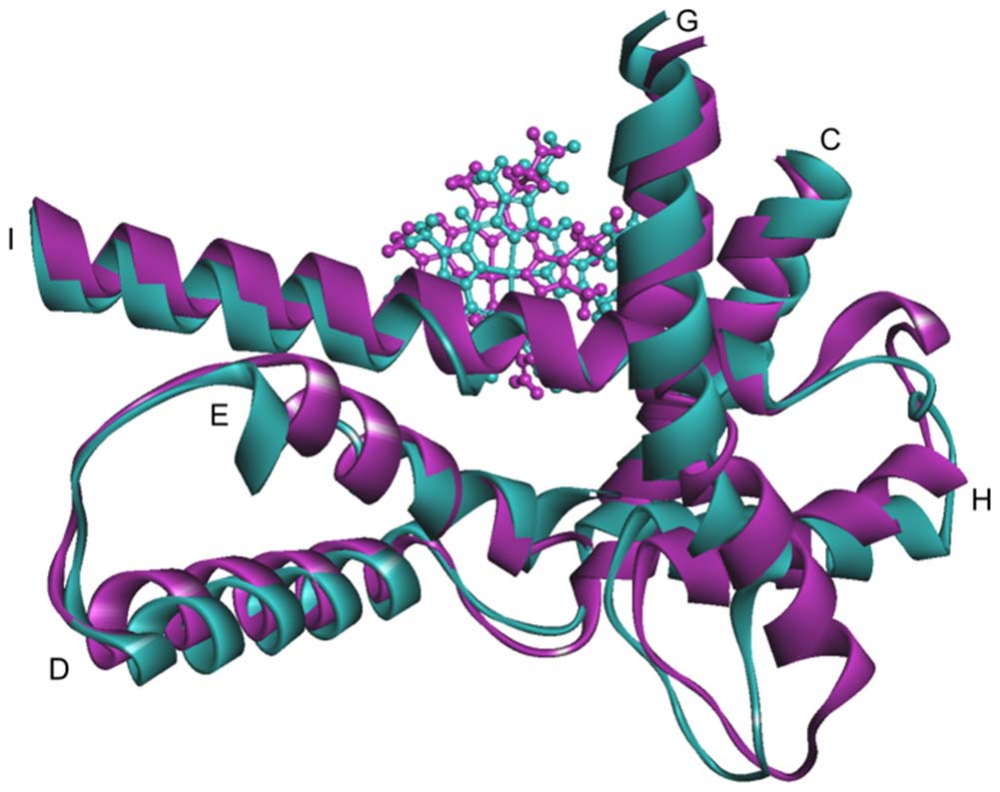
Structure of the helices around the heme. The C, D, E, G, H, and I helices of the CYP2B6 wild-type (blue) and CYP2B6.18 variant (purple) are shown by ribbons; the heme is shown by ball-and-stick representation.

**Figure 11:**
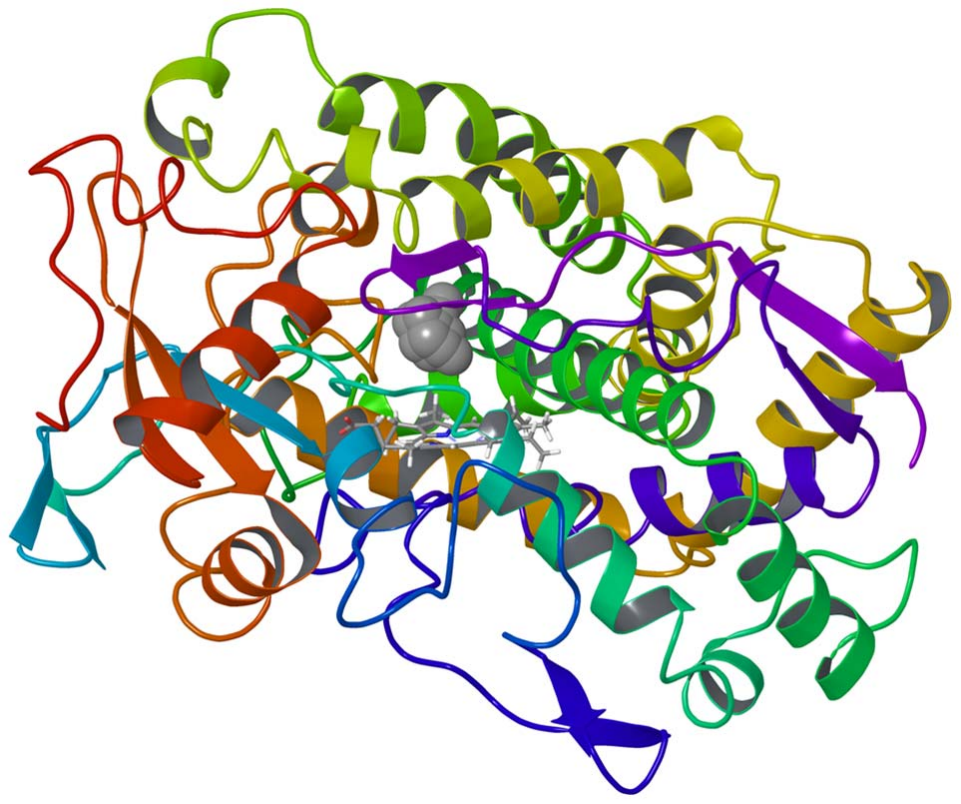
Ligand binding pocket of CYP2B6.18 detected by HBOP and HBSITE. The binding pocket is illustrated by probe spheres.

For CYP2B6.18, fewer docking poses were calculated, and no candidate was found for which the demethylation site of AM was close to the heme iron. The structure of CYP2B6.18 differed from that of the wild-type not only in the C and I helices, directly related to ligand and/or heme recognition, but also in the surrounding helices D, E, G, and H (Fig. 10). As mentioned above, some of these helices have an important role in enzyme activity and any structural change (such as in CYP2B6.18) may affect the recognition of AM and decrease the demethylation activity. For the 3D structure of CYP2B6.18 obtained with MD simulation, the ligand-binding pockets were detected with HBOP and HBSITE. Although the most probable binding pocket (HBS1) was located near the heme iron, the volume of the pocket was only 14 Å^3^. HBS1 of CYP2B6.18 is shown in Fig. 11, where HBS1 is illustrated by a collection of probe spheres. Because the volume of HBS1 for wild-type was 78 Å^3^, the mutation of CYP2B6.18 changed the shape and size of the ligand-binding pocket. The results indicate that the mutation of only one residue influences the location of helices and the movements of helices influence the ligand-binding pocket. Finally, no appropriate pose could be obtained for CYP2B6.11 and CYP2B6.21 by computational docking. Even the pose in which the methoxy group of AM is closest to heme iron was different from the predicted pose for wild-type, and all the predicted poses were inconsistent with the metabolic reaction of AM.

**Figure 12:**
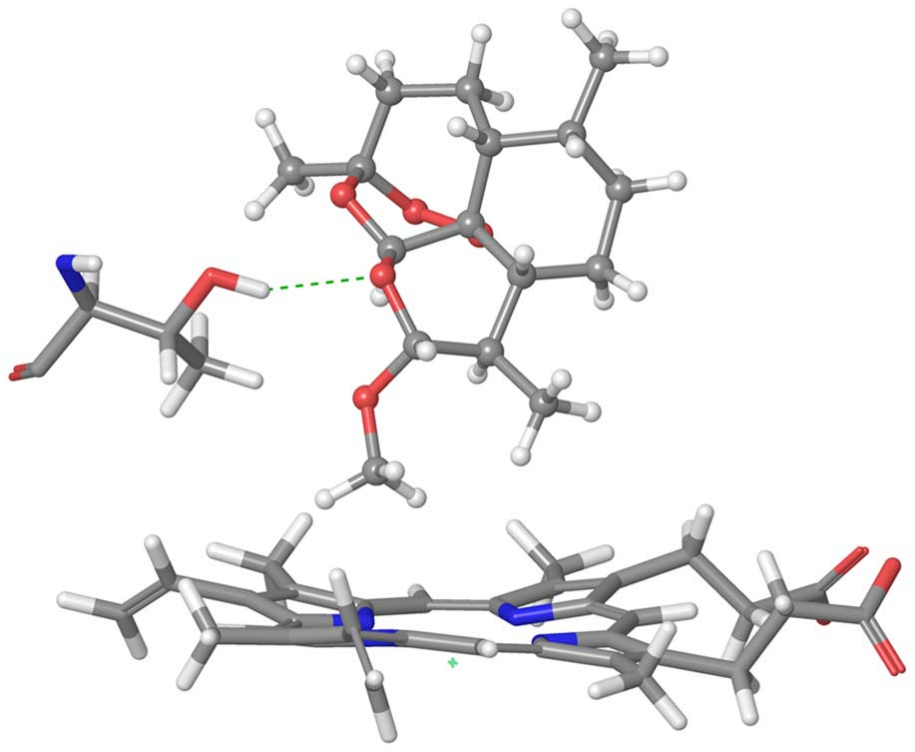
Hydrogen bond between AM and Thr302 in CYP2B6.8.

**Figure 13:**
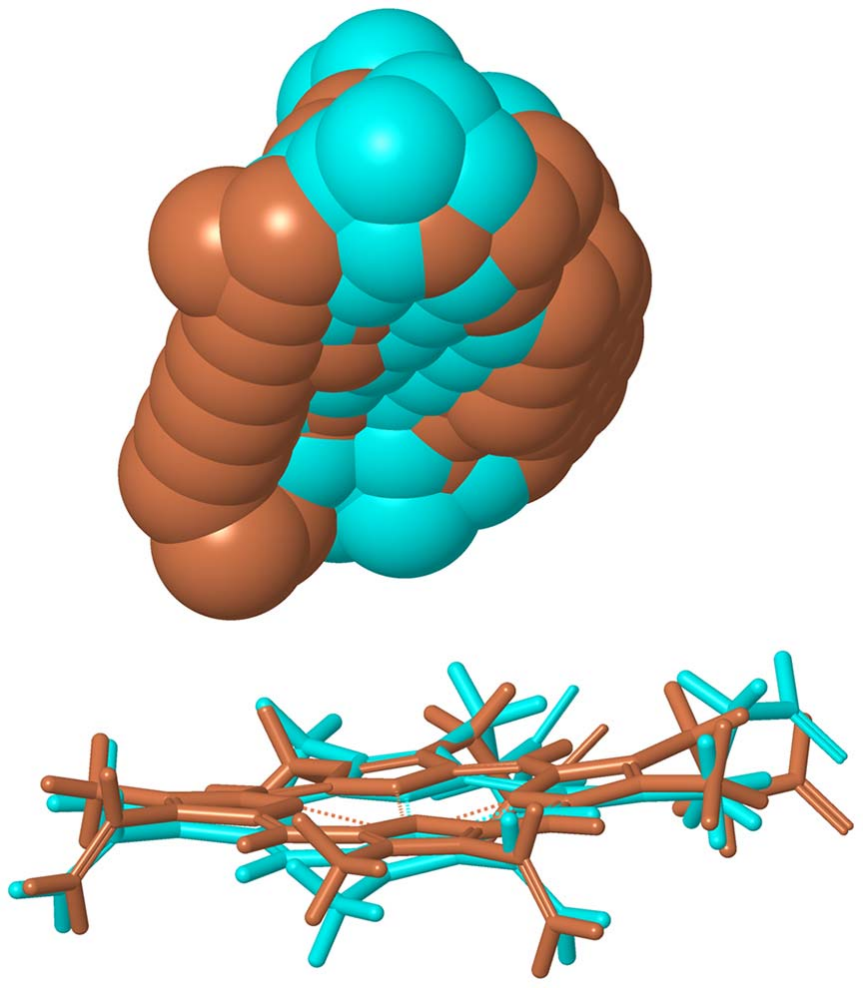
Ligand binding pockets of CYP2B6.1 (light blue) and CYP2B6.4 (brown). The pockets is illustrated by probe spheres.

Reasonable poses were obtained by the docking calculations for CYP2B6.4, CYP2B6.8, CYP2B6.12, CYP2B6.15, and CYP2B6.24. Because AM is the substrate of CYP2B6 and several processes are indispensable to catalytic reactions by CYP [3], the formation of a reasonable complex does not necessarily indicate that the mutant is active for AM demethylation. For example, even if the reasonable complex is formed, the change in the interaction between CYP and redox partner may cause a decrease or an absence of activity. However, the hydrogen bonding pattern in the complex between AM and some mutants were different from that of the wild-type. The hydrogen bond was formed between Thr302 and AM for CYP2B6.8 and CYP2B6.24; however, a hydrogen bond was not observed in the complex between Thr302 and AM for CYP2B6.1. Fig. 12 illustrates the hydrogen bond between AM and Thr302 in CYP2B6.8. Because the catalytic reaction of CYP includes several steps [3], the ligand of the heme iron is altered and the spin and oxidation states are changed in each step. Therefore, sufficient space to change the protein structure may be required for the catalytic reaction of CYP, and the additional hydrogen bond may prevent structural changes for CYP2B6.8 and CYP2B6.24. Although the hydrogen bond between Thr302 and AM was not observed in both CYP2B6.12 and CYP2B6.15, the RMSFs of these two mutants were largely different from that of the wild-type. The highest peak of RMSFs in CYP2B6.12 and CYP2B6.15 were different from that of the wild-type. Therefore, the decrease or absence of enzyme activity of these two mutants appears to be caused not by ligand recognition mechanisms but by protein flexibilities. In contrast, for the predicted structure of the complex between AM and CYP2B6.4, whose enzyme activity is higher than that of the wild-type, a hydrogen bond between Thr302 and AM was not observed, and CYP2B6.4 recognized AM by a binding mode similar to that for the wild-type. Fig. 13 illustrates the ligand-binding pockets for CYP2B6.1 and CYP2B6.4 detected with HBOP and HBSITE. The 3D structures of apoproteins refined with MD simulations were used for pocket detections, and the pockets were represented by collections of probe spheres; only HBS1 is shown in Fig. 13, where the wild-type is indicated in light blue and CYP2B6.4 is indicated in brown. Although the pocket for CYP2B6.4 was located in a position similar to the pocket for CYP2B6.1, the volume of the pocket was different (CYP2B6.1 = 78 Å^3^, CYP2B6.4 = 116 Å^3^). These results may indicate that the additional space at the active site plays an important role in the activity of CYP2B6.

The results obtained from MD simulation and docking studies are summarized in Table 1. “RMSF different from wild-type” in this table was checked if the difference of RMSFs between the mutant and wild-type was superior to 1 Å for at least one peak. The MD simulation of CYP2B6.4 converged at approximately 10 ns, and it had the highest peak of RMSF around Thr255 similar to the wild-type. Thus, the computational results for CYP2B6.4 and the wild-type are comparable in terms of both static and dynamic properties of the protein, which is consistent with experimental observations showing no decrease in the enzymatic activity of CYP2B6.4. These results are in favor of the use of these computational methods.

## Conclusions

We studied the influence of amino acid mutations on the properties of the metabolic enzyme CYP2B6 at the molecular and atomic level using MD simulations and docking studies. Our results suggest that a mutation not only changes the static structure of the enzyme but also the flexibility of the enzyme. The change in structural flexibility may in turn affect the enzyme activity. In particular, in the region from the G to the I helix, considered as the important region for CYP enzymatic activity, the structural flexibility plays a significant role in the activity. These changes of properties are occasionally due to a mutation of a remote residue; therefore, the whole protein structure must be investigated to clarify the influence of SNPs on pharmacokinetics. The methods used in this study (MD simulations of whole protein and docking studies between enzyme and substrate) could be useful for other CYPs and other drug-metabolizing enzymes.
